# Künstliche Intelligenz im Management der Anti-VEGF-Therapie: der „Vienna Fluid Monitor“ in der klinischen Praxis

**DOI:** 10.1007/s00347-022-01618-2

**Published:** 2022-04-14

**Authors:** P. Fuchs, L. Coulibaly, G. S. Reiter, U. Schmidt-Erfurth

**Affiliations:** 1 Vienna Clinical Trial Center (VTC), Universitätsklinik für Augenheilkunde und Optometrie, Medizinische Universität Wien, Währinger Gürtel 18–20, 1090 Wien, Österreich; 2 Christian Doppler Laboratory for Ophthalmic Image Analysis, Universitätsklinik für Augenheilkunde und Optometrie, Medizinische Universität Wien, Wien, Österreich

**Keywords:** Retinale Bildgebung, Neovaskuläre altersbedingte Makuladegeneration, Retinale Biomarker, Deep learning, Intravitreale operative Medikamentenapplikation, Retinal imaging, Neovascular age-related macular degeneration, Retinal biomarkers, Deep learning, Intravitreal injection

## Abstract

Der Vienna Fluid Monitor ist ein künstlicher Intelligenz(KI)-Algorithmus zur präzisen Lokalisation und Quantifizierung von retinaler Flüssigkeit. Der Algorithmus soll Klinikern und Klinikerinnen helfen, objektive und genaue Behandlungsentscheidungen bei der antivaskulären endothelialen Wachstumsfaktor(Anti-VEGF)-Therapie von Patienten mit neovaskulärer altersbedingter Makuladegeneration zu treffen. Ziel der Implementierung ist die Optimierung der Patientensicherheit, die Erhaltung der Sehleistung und gleichzeitig die Behandlungslast für das Gesundheitssystem und die Patienten zu verringern.

## Abstract (en)

The Vienna fluid monitor is an artificial intelligence (AI) algorithm for the precise localization and quantification of retinal fluid. The algorithm is designed to help clinicians to make objective and accurate decisions in the anti-vascular endothelial growth factor (anti-VEGF) therapy of patients with neovascular age-related macular degeneration. The goal of the implementation is to optimize patient safety, preserve visual function and simultaneously to reduce the treatment burden on the healthcare system and patients.

Künstliche Intelligenz (KI) ist nicht mehr aus dem Leben der meisten Menschen wegzudenken: selbstfahrende Autos, Sprachassistenten, mit denen man sich unterhalten kann, Webseiten, die in kürzester Zeit Texte in jegliche Sprachen übersetzen, Smartphones mit Gesichtserkennung, Navigationsgeräte, die in Echtzeit den Verkehrsstatus messen und vieles mehr. Auch in der Medizin hat die KI schon lange Fuß gefasst. Speziell im Bereich der Augenheilkunde erlebt sie gerade jetzt einen großen Auftakt.

Mittels optischer Kohärenztomographie (OCT) können mikrometergenaue dreidimensionale Aufnahmen der Retina gemacht werden. Diese Aufnahmen dauern nur wenige Sekunden, sind nichtinvasiv und ermöglichen dem Augenarzt und der Augenärztin eine schnelle Diagnosestellung. Durch die technischen Verbesserungen der OCT-Geräte und die steigende Zahl der Patienten und Patientinnen werden immer größere Datenmengen produziert, die kaum noch von menschlichen Untersuchern begutachtet werden können. Genau in diesem Zusammenhang kommt die KI ins Spiel. Diese kann die hochauflösenden OCT-Aufnahmen automatisch, schnell und objektiv verarbeiten. Hiermit wird für den Augenarzt und die Augenärztin eine effiziente und individuelle Beurteilung und Diagnostik verschiedener Netzhauterkrankungen geliefert.

Die KI kann Risiko und Krankheitsstadien verschiedener Netzhautpathologien anhand der OCT-Aufnahmen berechnen. Die ersten ophthalmologischen, auf KI basierenden Untersuchungsalgorithmen (IDx-DR, Digital Diagnostics, Coralville, IA, USA) sind bereits zugelassen und werden im Bereich des Screenings der diabetischen Retinopathie angewendet. Hierbei werden Fundusfotografien durchgeführt und das Risiko einer diabetischen Retinopathie wird bewertet. In Europa ist nun auch eine Zulassung für die automatische Erkennung und Messung von Flüssigkeit in der Netzhaut zugelassen worden (RetInSight, Wien, Österreich) [1].

Die Implementation der KI stellt eine Trendwende in der bildgebenden Diagnostik dar.

Mithilfe der KI sollen die Mediziner und Medizinerinnen bei der Bewältigung großer Mengen von Bildgebungsdaten unterstützt werden. Dies ermöglicht es ihnen, spezifische Merkmale zu erkennen und Diagnose- oder Therapiefehlern vorzubeugen. Zusätzlich kann die KI spezifische Krankheitsmuster identifizieren und mit noch unbekannten Merkmalen korrelieren, um neue Erkenntnisse zu gewinnen. Besonders im Bereich der altersbedingten Makuladegeneration (AMD) besteht bereits jetzt eine massive medizinische Unterversorgung abseits von klinischen Studien. Steigende Patientenzahlen und eine immer älter werdende Gesellschaft bedürfen eines effizienteren Diagnostik- und Managementprozesses. Ziel der KI ist es, die Sicherheit der Patienten zu gewährleisten, die Sehleistung zu erhalten und gleichzeitig die Behandlungslast für das Gesundheitssystem und die Patienten zu verringern.

## Was ist KI?

Die KI ist ein Teilgebiet der Informatik, welches darauf abzielt, Aspekte des menschlichen Denkens und Handelns mit intelligenten Maschinen nachzubauen. Ziel ist es Algorithmen zu programmieren, die eigenständig Probleme lösen. Der Begriff der künstlichen Intelligenz wurde von John Mccarthy geprägt. Dazu wurde 1956 der Dartmouth Workshop veranstaltet mit dem Ziel, „auf der Grundlage der Vermutung fortzufahren, dass jeder Aspekt des Lernens oder jedes andere Merkmal der Intelligenz im Prinzip so genau beschrieben werden kann, dass eine Maschine dazu gebracht werden kann, es zu simulieren“ [6]. Dieser Workshop gilt als die Geburtsstunde der künstlichen Intelligenz.

## Was ist maschinelles Lernen?

Maschinelles Lernen, ein Teilgebiet der KI, beruht auf der Generierung von Intelligenz und Wissen aus Erfahrung. Das maschinelle Lernen wurde von Arthur Samuel ins Leben gerufen. Es werden hierbei mathematische Modelle entwickelt, die verallgemeinerte Prinzipien aus vorhandenen Datensätzen erlernen und dann autonom an noch unbekannten Daten angewendet werden können.

## Was ist mehrschichtiges Lernen „deep learning“?

„Deep learning“ ist ein Teilbereich des maschinellen Lernens. Es basiert auf der Nutzung von neuronalen Netzwerken – sog. „artificial neural networks“ (ANN). Diese Netzwerke beruhen auf der Imitation von neuronalen Strukturen des menschlichen Nervensystems. Die ANN sind ein Rechensystem, das aus nacheinander geschalteten künstlichen Neuronen besteht. Es ist in Schichten organisiert und kann wie das menschliche Gehirn Muster erkennen und verarbeiten. ANN fanden schnell ihre Verwendung in den 90er-Jahren. Es konnte bewiesen werden, dass sie Myokardinfarkte in Notaufnahmen, renale Tumoren in Ultraschalluntersuchungen und diabetische Retinopathien in Fundusfotos im Vergleich zu menschlichen ExpertInnen genauso gut erkennen konnten.

Aus den ANN entwickelten sich sog. „deep neural networks“ (DNN). Ein DNN ist ein ANN mit mehreren Zwischenschichten und weniger künstlichen Neuronen. Der wesentliche Vorteil der DNN ist, dass sich die Leistung mit einem wachsenden Datensatz kontinuierlich verbessert. Zusätzliche profitierten DNN durch Verbesserungen der Rechenleistung der Computer, welche zu einer schnelleren Datenverarbeitung führten und alle anderen Formen des maschinellen Lernens übertrafen.

## KI im Bereich des Patientenmanagements von AMD-Patienten

Die Einführung der Anti-VEGF-Therapie in der Behandlung retinaler Erkrankungen hat den Alltag der Augenkliniken maßgeblich verändert. Die Behandlung ist hochwirksam und wurde binnen kurzer Zeit nicht nur zum häufigsten operativen Eingriff in der Augenheilkunde, sondern in der gesamten Medizin. Während der therapeutische Nutzen bei Krankheitsbildern wie der neovaskulären altersbedingten Makuladegeneration (nAMD), retinaler Venenverschlüsse oder dem diabetischen Makulaödem unumstritten ist, bleiben intravitreale Injektionen ein invasiver Eingriff mit seltenen, aber teils schweren Komplikationen [4]. Der chronische Charakter retinaler Erkrankungen erfordert regelmäßige Wiederbehandlungen. Dies und die stetig wachsenden Patientenzahlen stellen eine nicht zu vernachlässigende logistische wie auch finanzielle Belastung für das Krankenhauspersonal und das Gesundheitssystem dar [5]. Um die Anti-VEGF-Therapie zu optimieren, kann KI effizient und zuverlässig eingebunden werden. So kann trotz steigender Injektionszahlen eine objektive und individuelle Behandlung für jeden Patienten ermöglicht werden [3, 4]. Im täglichen Klinikalltag beruhen Wiederbehandlungsentscheidungen in der Anti-VEGF-Therapie auf der Beurteilung der auf OCT-Bildern erkennbaren pathologischen Biomarker. Insbesondere exsudative Prozesse in Form von subretinaler Flüssigkeit (SRF) und intraretinaler Flüssigkeit (IRF) spielen eine entscheidende Rolle [9]. Die Behandlungsentscheidung basiert dabei jedoch primär auf einer qualitativen („vorhanden“ oder „nicht vorhanden“) Interpretation durch den behandelnden Augenarzt oder die behandelnde Augenärztin. Damit sind Behandlungsoptionen in vielen Fällen nicht objektiv nachvollziehbar und aufgrund ihres subjektiven Charakters anfällig für Fehler. Ungenauigkeiten dieser Art könnten eine Erklärung für die geringeren Therapieerfolge in der „Real-World“ im Vergleich zu klinischen Studien erklären [6].

Eine quantitative Vermessung der vorhandenen Flüssigkeit führt hingegen zu einer präzisen, objektiven und nachvollziehbaren Wiederbehandlungskriterien. Eine manuelle, quantitative Flüssigkeitsvermessung ist im klinischen Alltag aufgrund des damit verbundenen hohen Zeitaufwandes nicht umsetzbar [7, 8]. Die Einbindung von KI-Algorithmen in die Beurteilung von OCT-Bildern führt zu einem Paradigmenwechsel in der Anti-VEGF Behandlung. Mit der Unterstützung von KI können digitale Bilder wie auch das in der Augenheilkunde eingesetzte OCT schnell und umfassend analysiert werden.

## Der Vienna Fluid Monitor

**Figure 1:**
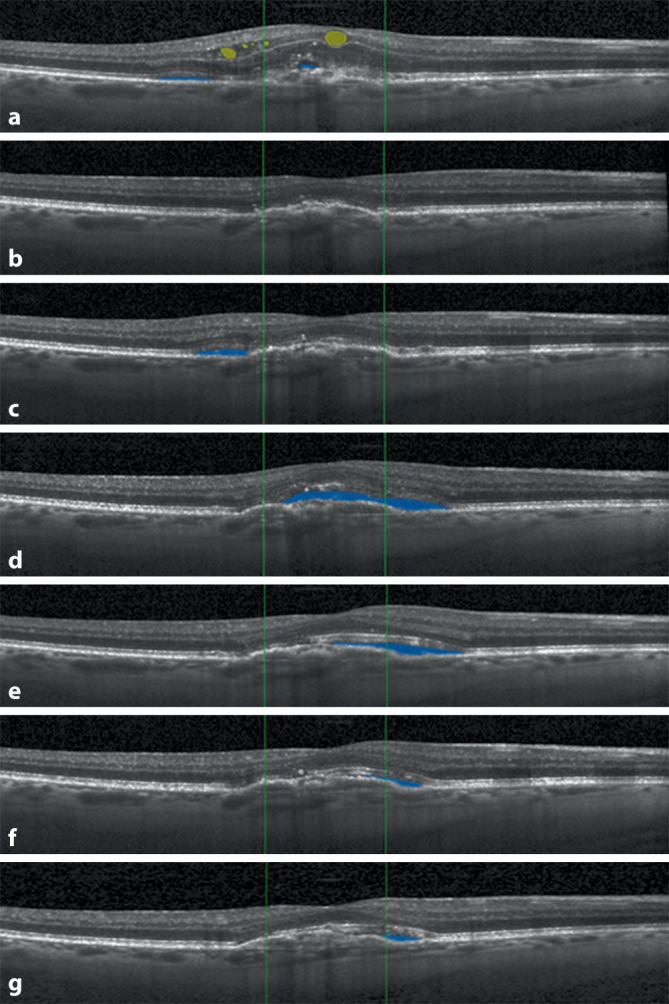

**Figure 2:**
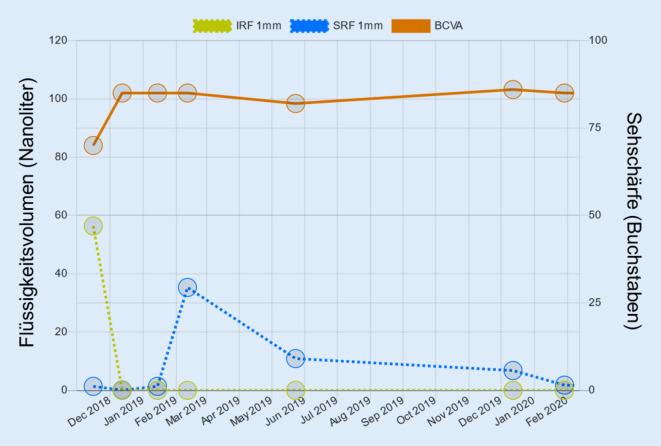

Ein auf „deep learning“ basierender KI-Algorithmus, der retinale Flüssigkeit präzise (pro Bildpunkt) lokalisieren und quantifizieren kann, wurde an der Universitätsklinik für Augenheilkunde und Optometrie der Medizinischen Universität Wien entwickelt. Der sog. „Vienna Fluid Monitor“ (RetInSight, Wien, Österreich) wurde an großen und vielfältigen Datensätzen mit an nAMD erkrankten Patienten trainiert und anhand von Befunden aus der klinischen Routine (VIBES, Medizinische Universität Wien von 2007 bis 2018) validiert. Der Fluid Monitor-Algorithmus wurde nicht nur auf Studiendaten, sondern auch auf OCT-Bildern aus der Routinebehandlung validiert. Der Vergleich der Patientenkollektive als auch der Vergleich zwischen der automatischen und der Annotation des humanen Experten zeigten dabei keinerlei Unterschiede, d. h. dass das Tool ohne Einschränkung in der klinischen Praxis anwendbar ist. Dabei werden die OCT-Bilder in einen Cloudspeicher hochgeladen und innerhalb kürzester Zeit automatisiert ausgewertet. Der Algorithmus unterscheidet zwischen SRF und IRF, und die Flüssigkeitsmengen werden nach vollständiger Analyse in Nanoliter angezeigt und auf den OCT-Bildern farblich visualisiert (Abb. 1). Die genaue Flüssigkeitsdynamik kann im Verlauf abgebildet werden (Abb. 2). Der Algorithmus unterscheidet aktive und nichtaktive, sog. degenerative Zysten anhand der statischen Morphologie und Menge mit fehlenden Fluktuationen unter Therapie. Die präzise Quantifizierung von Flüssigkeiten intraretinal sowie subretinal erlaubt entsprechend auch außerordentlich verlässlich die Unterscheidung zwischen dynamischem exsudativem Fluid im Vergleich zu statischem, degenerativem Fluidpooling. Da der Algorithmus speziell auf definierten Krankheitsbildern, z. B. der neovaskulären AMD, trainiert und validiert ist, unterscheidet er zuverlässig zwischen intraretinaler Flüssigkeit und sog. „outer retinal tubulations“ oder Kavitationen. Die rezente Literatur zeigt eindrücklich, dass dynamische Fluktuationen des Fluids Ursache für den Visusverlust sind, und gerade diese werden präzise messbar und entsprechend gezielt behandelbar [2]. Segmentierungsartefakte bei ausreichender Bildqualität treten nicht auf, können aber vom beurteilenden Arzt jederzeit verworfen werden.

Der Algorithmus ist nicht geräte- oder herstellerspezifisch. In Bezug auf die Gerätespezifität gilt, dass jeder Algorithmus gerätespezifisch trainiert und validiert werden muss. Der Fluid Monitor wird im April 2022 für das Heidelberg Spectralis OCT (Heidelberg Engineering, Heidelberg, Deutschland) gelauncht und anschließend für die üblichen Zeiss- (Carl Zeiss Meditec, Dublin, CA, USA) und Topcon- (Topcon Corp, Itabashi, Japan) OCT-Geräte. Es sind keine speziellen OCT-Protokolle einzuhalten. Naturgemäß gilt, dass ein dichteres Scan-Pattern zu einer höheren Auflösung führt. Der Einsatz des Fluid Monitors bei Real-World-Patienten hat gezeigt, dass ein Routinebild uneingeschränkt präzise ausgewertet werden kann. Der Fluid Monitor ist ein CDSS, d. h. ein „clinical decision support system“, das den Arzt in seiner Tätigkeit unterstützt. Dies geschieht beim Fluid Monitor durch eine präzise Identifikation, Lokalisation und Quantifizierung von retinaler Flüssigkeit, bei der Beurteilung der Erkrankungsaktivität und des Therapieansprechens. Es wird keinerlei Therapieempfehlung gegeben, sondern es handelt sich um eine Auswertung des Flüssigkeitsbefundes. Der Algorithmus ersetzt entsprechend auch nicht die fachärztliche Diagnose der zugrunde liegenden Erkrankung. Mithilfe des Vienna Fluid Monitors konnten quantitative Flüssigkeitsanalysen klinischer Studien (HARBOR, HAWK/HARRIER, FLUID, TREND) durchgeführt werden. Zudem wurden 24.473 Cirrus- (Carl Zeiss Meditec, Dublin, CA, USA) und 13.822 Spectralis- (Heidelberg Engineering, Heidelberg, Germany) OCT-Aufnahmen von Patienten mit nAMD aus der klinischen Routine analysiert (VIBES). Durch die quantitative Analyse dieser Auswertung von exsudativen Prozessen bei der nAMD konnten wertvolle neue Informationen zur Erkrankung gewonnen werden. Auch die „Structure/Function“-Korrelation zwischen Flüssigkeit und Sehleistung konnte präziser untersucht werden. Vor allem IRF-Volumina korrelieren signifikant mit der Sehleistung und beschreiben die Sehleistung besser als die zentrale Netzhautdicke [7, 9].

In einem weiteren Schritt wird der Vienna Fluid Monitor in einer prospektiven, multizentrischen Studie eingesetzt. Dabei werden sowohl vorbehandelte als auch unbehandelte nAMD-Patienten aus der klinischen Routine eingeschlossen. Die Studienpopulation entspricht somit der „Real-World“-Situation. Der Vienna Fluid Monitor unterstützt behandelnde Ärzte und Ärztinnen bei der Entscheidung einer Wiederbehandlung von exsudativen Prozessen der nAMD. Wiederbehandlungen sollen an die individuelle Krankheitsdynamik angepasst werden. Dies ermöglicht eine deutliche Verbesserung der „Point-of-care“-Behandlung. Die große Anzahl an Informationen, die durch die OCT-Bildgebung generiert werden, kann effektiv genutzt werden und trägt maßgeblich zu einer Personalisierung der intravitrealen Anti-VEGF-Therapie bei. Der Vienna Fluid Monitor ist somit das erste klinische AI-Tool im Management der intravitrealen Therapie. Er ermöglicht der behandelnden Ärztin und dem behandelnden Arzt eine objektive, präzise sowie zeitsparende Bewertung des Therapieeffekts der intravitrealen Behandlung in Echtzeit. Durch die Verwendung von KI-Algorithmen wird eine Präzisionsmedizin in der Ophthalmologie für jeden Patienten ermöglicht und ein wichtiges Werkzeug in der Behandlung retinaler Erkrankungen in die klinische Routine integriert.

## Fazit für die Praxis

- Der Vienna Fluid Monitor ist das erste klinische künstliche Intelligenz(KI)-Tool im Management der intravitrealen Therapie.
- Implementierung von KI lohnt sich besonders im Bereich der Anti-VEGF-Therapie von Patienten mit neovaskulärer AMD.
- Der Vienna Fluid Monitor ermöglicht die genaue Lokalisierung und Quantifizierung von Flüssigkeit in der Netzhaut.
- Durch KI wird eine objektive, zeitsparende und individuelle Präzisionsmedizin ermöglicht.
